# Evaluation of allograft decontamination with two different antibiotic cocktails at the Treviso Tissue Bank Foundation

**DOI:** 10.1371/journal.pone.0201792

**Published:** 2018-08-02

**Authors:** Adolfo Paolin, Lisa Spagnol, Giuseppe Battistella, Diletta Trojan

**Affiliations:** 1 Fondazione Banca dei Tessuti di Treviso, Treviso, Italy; 2 Innovation, Development and Planning Department, Ca’ Foncello Hospital, Treviso, Italy; University of Toledo, UNITED STATES

## Abstract

Microbiological contamination of retrieved tissues is a critical aspect of allograft safety and tissue banks must continuously implement decontamination procedures to minimize tissue contamination. In this study we compared the decontamination efficacy of a new antibiotic cocktail (cocktail B: BASE medium with Gentamicin, Meropenem and Vancomycin) with the cocktail previously adopted at Treviso Tissue Bank Foundation (FBTV) (cocktail A: RPMI medium with Ceftazidime, Lincomycin, Polymyxin B and Vancomycin). Two decontamination steps were carried out, the first immediately after retrieval, the second after processing. The contamination rate was calculated before processing (Time 1) and cryopreservation (Time 2) for total tissues, musculoskeletal tissues and cardiovascular tissues, and the bacterial species involved were analyzed. Cocktail A was used to decontaminate 3548 tissues, of which 266 were cardiovascular and 3282 musculoskeletal tissues. For cocktail A, total tissue contamination was 18.6% at Time 1 and 0.9% at Time 2, with 15.7% contaminated musculoskeletal tissues at Time 1 and 0.4% at Time 2, respectively, while cardiovascular tissues were 50% contaminated at Time 1 and 6.4% at Time 2. Cocktail B was used to decontaminate 3634 tissues of which 318 were cardiovascular and 3316 musculoskeletal tissues. For cocktail B, total tissue contamination was 8.6% at Time 1 and 0.2% at Time 2, with 7.6% contaminated musculoskeletal tissues at Time 1 and 0.03% at Time 2, respectively. Contamination of cardiovascular tissues was 20.4% at Time 1 and 1.9% at Time 2. Intergroup and intragroup contamination rates decreased statistically significantly (p<0.05). Our results have shown that cocktail B was more effective than cocktail A in killing bacteria in both cardiovascular and musculoskeletal tissues during the two decontamination cycles.

## Introduction

Tissue banking consists of processing, decontaminating and preserving homografts harvested from donors for future clinical applications. Transmission of infection via transplantation of allografts, including solid organs, eyes, and tissues, is an uncommon but potentially life-threatening event [1]. Allografts are exposed to potential contamination during procurement, processing and preservation, representing a potential risk for transmission of pathogenic microorganisms [2–4]. Different tissues may experience different rates of contamination. Contamination rates are usually higher for cardiovascular tissues (CVT) than for musculoskeletal tissues (MST), and skin commensals, as *coagulase negative Staphylococci*, are the most commonly isolated organisms [5–9]. Every tissue bank performs decontamination procedures to overcome the problem of bacterial contamination. Microbiological decontamination techniques include chemical and antibiotic treatments [10–12] or terminal sterilization methods to target microorganisms [13–14]. European Directive 2006/86/EC requires that tissue banks specify, document and validate a microbial inactivation procedure for the decontamination of donor tissues [15]. Focusing on antibiotic treatments, tissue banks adopt cocktail solutions of various compounds with antimicrobial activity. However, the choice of antimicrobial agents, their concentration in the cocktail, the temperature at which they are used and the length of exposure of tissue to the agents differ widely from bank to bank [16–17]. Antibiotics like vancomycin, b-lactams, polymyxin and gentamicin are the most widely used to inhibit the growth of the most representative bacterial species isolated from the skin and intestinal flora [18–19]. Currently, however, no standard decontamination approach is universally adopted by all tissue banks and no consensus exists on an optimal antibiotic cocktail. Treviso Tissue Bank Foundation (FBTV) identified the need to develop and evaluate a new quality pathway to address the problem of microbial resistance to antibiotics in relation to the composition of the bacterial flora contaminating banked tissues. Accordingly, an extensive analysis of contamination rates for all tissue types was carried out with the purpose of validating a more effective decontamination procedure. FBTV proceeded as follows: after carefully assessing the species usually isolated in tissues and the level of tissue contamination during each tissue banking specification phase, the facility tested four new antibiotic (AB) cocktails composed of various antibiotics at different temperatures. The most efficient cocktail was then introduced into bank practice. The aim of this 2-year observational study was to describe the results of microbiological cultures submitted to longitudinal analysis to compare the tissue contamination rate and decontamination efficacy of two antibiotic cocktails, i.e. cocktail B, currently used at FBTV following *in vitro* validation [17], and the previously adopted cocktail A.

## Materials and methods

### Tissues and data collection

Bacterial contamination was analyzed in 7182 tissues, of which 3548 were decontaminated with cocktail A (Group A) and 3634 with cocktail B (Group B), over a period of two years, i.e. one complete year for each group. The longitudinal contamination profile was defined for each individual allograft. Tissues discarded post retrieval as unsuitable for clinical use due to morphological abnormalities or because the donor was positive for one of the relevant serological markers, were excluded. The tissues included in this survey account for 55% and 93% of all retrieved CVT and MST, respectively. Tissues were harvested after organ retrieval in heart-beating donors (HBD), and within 24h of cardiac arrest in non-heart-beating donors (NHBD), by our retrieval team, in operating rooms using standard aseptic techniques. NHBD accounted for approximately 85% of the donations, while the remaining 15% were HBD. Prior to tissue retrieval the skin was surgically scrubbed with chlorhexidine solution and shaved, followed by an additional application of chlorhexidine and povidone iodine. Immediately after retrieval, tissues were rinsed with isotonic saline solution. Once rinsing was complete, the tissues were placed in a sterile container filled with the antibiotic solution and kept at +4°C until the start of processing at the FBTV facilities.

The study was exempt from the need for ethics committee review due to the nature of the analysis, i.e., the performance of microbiological tests on samples of rinsing solutions after tissue decontamination.

### Antibiotic cocktail formulations and decontamination procedure

This study included two antibiotic cocktail formulations. Cocktail A was composed of RPMI medium supplemented with Ceftazidime (240μg/ml) (Fresenius-Kabi, Isola della Scala, Verona, Italy), Lincomycin (120μg/ml), Polymyxin B (100μg/ml) (Biochrom, Milano, Italy) and Vancomycin (50μg/ml) (Hospira, Napoli, Italy), as implemented by the European Homograft Bank at the time of adoption by FBTV.

Cocktail B was composed of BASE medium (Alchimia, Ponte San Nicolò, Padova, Italy) supplemented with Gentamicin sulfate (200 μg/ml) (Fisiopharma, Palomonte, SA, Italy), Meropenem (200 μg/ml) (Hikma Italia, Pavia, Italy), and Vancomycin (100 μg/ml) (Pharmatex, Milan, Italy). Polymyxin B was not included as a potential AB in the new cocktail formulation, partly on account of its programmed withdrawal in Italy as a systemic antibiotic, due to toxicity, and its restriction to topical applications.

The decontamination procedure consisted of two identical decontaminating steps: the first after retrieval and before the processing phase; the second after processing and before the freezing/ cryopreservation phase. During each decontamination step, the whole allograft was immersed in the antibiotic solution (cocktail A or B) in a single sterile collection container and kept at +4°C for at least 48h. Each step was carried out in class A biohazard laminar airflow cabinets with a class B environment.

### Microbiological analysis

Bacteriological examinations for aerobic and anaerobic bacteria and fungi/yeasts were performed after each decontamination step, i.e. before processing (Time 1) and before freezing/ cryopreservation (Time 2). Tissues were rinsed with isotonic saline after each decontamination step and samples of the rinsing solution of each tissue were collected, without filtering, for microbiological analyses. All procedures were carried out at room temperature. To detect microbial growth, samples were inoculated and incubated in culture vials of BD BACTEC™/Alert Fluorescent Test Technology, in accordance with the manufacturer’s instructions (BD, Becton, Dickinson and Company, New Jersey). Where present, the microorganisms in the BACTEC vial metabolized the substrates, producing CO_2_. The increased fluorescence caused by higher amounts of CO_2_ was detected by the BACTEC fluorescent series instrument. The instrument analyzed the amount and rate of CO_2_ increase to determine if the vial was positive, i.e., whether the test sample contained viable organisms. If it tested positive after 7 days, the microorganisms were isolated and identified using standard procedures. Microbiological cultures and analyses were carried out by an accredited in-hospital microbiology laboratory and interpreted by a microbiologist with specific expertise. In compliance with our policy, the following species were classified as non-compliers: *Clostridium spp*., *Fungi/Yeast*, *Mycobacteria*, *Streptococcus pyogenes*, *Streptococcus pneumoniae*, *Pseudomonas aeruginosa*, *Serratia marcescens*, *and Meningococcus*. Whenever any of these species were isolated, the tissue was discarded regardless of the step at which positivity was detected. In addition, all tissues found positive after the second decontamination were also discarded.

### Statistical analysis

Data were analyzed using software version WinPEPI 11.65. “N-1” chi-square tests for independent variables [20] were used. To quantify uncertainty, 95% confidence intervals were also calculated by traditional (log-transformation) method. *P* value< 0.05 was considered statistically significant.

## Results

### Tissue characteristics

**Fig 1:**
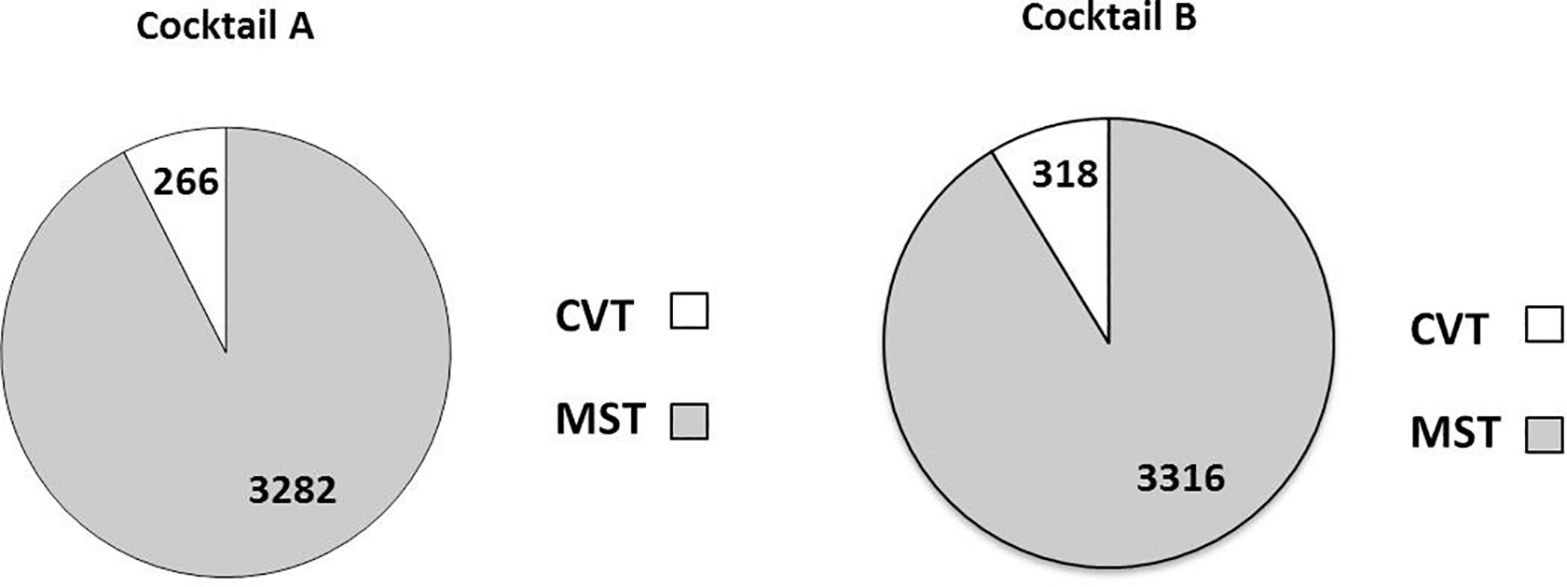
Tissue data.

A total of 7182 MST and CVT were eligible for the study. 3548 tissues (266 CVT and 3282 MST) belonged to Group A while 3634 tissues (318 CVT and 3316 MST) belonged to Group B (Fig 1).

### Contamination at Time 1

The results of tissue contamination at Time 1 are reported in Table 1.

**Table 1:** No. of tissues contaminated in Groups A and B at the two time points.

	Time 1		Time 2	
	Contaminated tissue (%)		Contaminated tissue (%)	
	Group A	Group B	Group A	Group B
MST	15.7%	7.6%	0.4%	0.03%
CVT	50%	20.4%	6.4%	1.9%

% are expressed as the correlation between the numbers of total MST and CVT

Out of the 3548 tissues of Group A, 649 proved positive at Time 1 [18.2% (95% CI 17.07–19.59)], while 316 of the 3634 tissues of Group B tested positive [8.6% (95% CI 7.81–9.65)] (*p*<0.001). In Group A, 515 of the 649 positive tissues were MST (79.3% of the contaminated tissues) and 134 were CVT (20.7% of the contaminated tissues), while 251 of the 316 positive tissues in Group B were MST (79.4% of the contaminated tissues) and 65 were CVT (20.6% of the contaminated tissues). The percentage of the total MST testing positive decreased significantly from 15.7% (95% CI 14.48–16.97) in Group A to 7.6% (95% CI 6.71–8.51) in Group B (*p* <0.001), while the percentage of the total CVT testing positive dropped significantly from 50% (95% CI 44.38–56.36) of Group A to 20.4% (IC95 16.28–25.14) of Group B (*p* <0.001).

### Contamination at Time 2

The contamination results at Time 2 are reported in Table 1.

A total of 29 out of the 3548 tissues proved positive in Group A [0.8%, (95% CI 0.56–1.16)] and a total of 7 tissues of the 3634 tissues tested positive in Group B [0.2% (95% CI 0.08–0.38)] (*p* <0.001). Of the 29 positive Group A tissues, 12 were MST and 17 were CVT, while in Group B, 1 was MST and 6 were CVT. The percentage of the total MST testing positive differed significantly, decreasing from 0.4% (95% CI 0.20–0.62) in Group A to 0.03% (95% CI 0.002–0.149) in Group B (*p* = 0.002), while the percentage of total positive CVT dropped from 6.4% (95% CI 3.89–9.84) in Group A to 1.9% (95% CI 0.77–3.88) in Group B (*p* = 0.005).

### Contaminating species

The contaminating species for MST and CVT for both groups, at Times 1 and 2, are listed in Tables 2 and 3, respectively.

**Table 2:** Species isolated at Times 1 and 2 for MST, in both groups.

**Time 1**					
**Group A**			**Group B**		
**Microorganism**	**No. of tissues**	**%**	**Microorganism**	**No. of tissues**	**%**
*Coagulase -Staphylococcus*	365	70.9	*Coagulase—Staphylococcus*	176	70.1
*Staphylococcus aureus*	28	5.4	*Staphylococcus aureus*	5	2.0
*Enterococcus spp*	23	4.5	*Enterococcus spp*	2	0.8
*Haemophilus parainfluenzae*	23	4.5	*Streptococcus spp*	12	4.8
*Streptococcus spp*	16	3.1	*Bacillus spp*	10	3.9
*Bacillus spp*	16	3.1	*Clostridium spp*	14	5.6
*Clostridium spp*	15	2.9	*Micrococcus luteus*	4	1.6
*Micrococcus luteus*	12	2.3	*Bacteroides spp*	6	2.4
*Bacteroides spp*	8	1.6	*Escherichia coli*	1	0.4
*Pseudomonas aeruginosa*	4	0.8	*Other (6 genera)*	21	8.4
*Escherichia coli*	3	0.6			
*Other (2 genera)*	2	0.4			
***Total tissues***	**515**		***Total tissues***	**251**	
**Time 2**					
**Microorganism**	**No. of tissues**		**Microorganism**	**No. of tissues**	
*Coagulase -Staphylococcus*	6		*Staphylococcus epidermidis*	1	
*Streptococcus spp*	5				
*Bacillus simplex*	1				
***Total tissues***	**12**		***Total tissues***	**1**	

**Table 3:** Species isolated at Time 1 and Time 2 for CVT in both groups.

**Time 1**					
**Group A**			**Group B**		
**Microorganism**	**No. of tissues**	**%**	**Microorganism**	**No. of tissues**	**%**
*Coagulase -Staphylococcus*	60	44.7	*Coagulase -Staphylococcus*	16	24.6
*Staphylococcus aureus*	9	6.7	*Staphylococcus aureus*	4	6.1
*Streptococcus spp*	20	14.9	*Streptococcus spp*	19	29.2
*Clostridium spp*	10	7.4	*Clostridium spp*	5	7.7
*Granulicatella adiacens*	7	5.2	*Enterococcus spp*	3	4.6
*Enterococcus spp*	4	3.0	*Bacillus spp*	4	6.1
*Peptostreptococcus anaerobius*	4	3.0	*Others (8 genera)*	14	21.5
*Others (10 genera)*	20	14.9			
***Total tissues***	**134**		***Total tissues***	**65**	
**Time 2**					
**Microorganism**	**No. of tissues**		**Microorganism**	**No. of tissues**	
*Clostridium spp*	4		*Streptococcus anginosus*	1	
*Granulicatella adiacens*	3		*Staphylococcus hominis*	3	
*Streptococcus spp*	2		*Candida albicans*	1	
*Staphylococcus aureus*	2		*Saccharomyces rosei*	1	
*Staphylococcus lugdunensis*	1				
*Gemella morbillorum*	2				
*Enterococcus gallinarum*	1				
*Bacteroides ovatus*	1				
*Lactococcus fermentum*	1				
***Total tissues***	**17**		***Total tissues***	**6**	

### MST

At Time 1, *coagulase-negative Staphylococci* were the most commonly isolated microorganisms in both groups (70.8% and 70.1% of the positive cultures in Groups A and B, respectively), followed by several genera whose detection and percentages differed in the two groups (Table 2). At Time 2, there were 12 positive tissues in Group A but only 1 in Group B. At Time 1, 454 out of 515 tissues in Group A were positive for only one species (88.2%), while the remaining 61 tissues (11.8%) were contaminated with 2 to 4 species, with 13 genera in total. In Group B, 187 out of 251 tissues (74.5%) were positive for only 1 species while the remaining 64 tissues (25.5%) were contaminated with 2 to 4 species. At Time 2, only 1 species/per tissue was isolated with 3 and 1 genera in total for Groups A and B, respectively.

### CVT

At Time 1, the most commonly isolated species in CVT, as in MST, were *coagulase negative Staphylococci*, but at much lower percentages (44.7% and 24.6% of the total contamination in Groups A and B, respectively). The remaining contaminations were due to a higher number of genera than observed in MST, and there was overlap between the two groups, but with different percentages among the various species (Table 3). At Time 1, in Group A, 88 out of 134 positive tissues were positive for only one species (65.6%), while the remaining 46 tissues (34.4%) were contaminated with 2 to 4 species, with 17 genera in total. In Group B, 43 out of 65 (66.2%) positive tissues were contaminated with only one species, while the remaining 22 tissues (33.8%) were positive for 2 to 4 species, with 20 genera in total. At Time 2, 17 species were detected in Group A and 6 in Group B, and only one species per tissue was isolated, with 9 and 4 genera in total for Groups A and B, respectively.

## Discussion

All tissue banks share the common objective of eradicating any pathogens present in tissues, to prevent the risk of transmitting potentially infectious agents to recipients [21–25] and reduce the number of tissues discarded for being positive for post decontamination residues. For many years, FBTV adopted a decontamination protocol which failed to totally eradicate the contaminants, resulting in the discard of large quantities of tissues, particularly of CVT origin. This prompted the need to develop a new cocktail that was optimally effective, at the previously adopted temperatures and exposure times [17], against species historically isolated at our facility, with the exception of tissues contaminated with non-compliers, which were discarded regardless of the step in the procedure at which positivity was detected. After validation of cocktail B *in vitro*, we conducted an extensive comparative analysis of the microbiological results of tissues decontaminated with cocktail B and cocktail A, over the course of one year, to evaluate whether it was also more effective in practice. Cocktail A was used to decontaminate 3548 tissues, of which 266 were CVT and 3282 MST, while cocktail B was used to decontaminate 3634 tissues, of which 318 were CVT and 3316 MST. Before processing and storage, the contamination rate was calculated for MST, CVT, and total tissues, and the bacterial species involved were analyzed. Our data highlight that cocktail B was more effective than cocktail A in both decontamination steps and for both types of tissue. In the first decontamination step with cocktail B, there was an overall 50% decrease in the rate of contaminated tissues compared to cocktail A, with global contamination falling from 18.2% to 8.6%. Specifically, total contamination decreased from 15.7% to 7.8% for MST and from 50% to 20.4% for CVT. A much higher percentage of MST than of CVT were contaminated by a single species, in both groups. The results of the first decontamination cycle with cocktail A were comparable with earlier results published on tissue decontamination at our bank over a 4-year period, for both MST and CVT [26]. They also corroborated the findings of other authors that skin commensals [6, 27–31] and intestinal and respiratory tract flora [26,29,32] were the most commonly identified species in cadaveric tissue donors. In keeping with previous literature on tissue contamination, our results confirmed that a single decontamination step was not sufficient to eradicate all pathogens. This prompted the need for a second decontamination, also taking into account that unclassified species among the non-compliers, which could potentially be eradicated by the antibiotic cocktail, were the main source of residual contamination. After the second decontamination step, cocktail B reduced the percentage of positive tissues far more than cocktail A, resulting in <2% and practically zero residual positivity (only 1 positive tissue) for CVT and MST, respectively, while cocktail A left higher percentages of positive tissues, in keeping with previous reports [26]. Ultimately, a lower number of both MST and CVT tissues tested positive post decontamination with cocktail B than with cocktail A. The number of species isolated per tissue was confirmed to be greater than one, particularly in CVT, and this ratio did not significantly change compared to the first decontamination for either cocktail, despite the lower number of positive tissues. There may be several explanations for the residual percentage of contaminated tissues at the end of the process. Multiple contaminations, which are higher in CVT, might explain the incomplete decontamination achieved in this type of tissue. This hypothesis is supported by the finding that MST, contaminated in nearly 90% of cases by a single species, were almost completely decontaminated at the end of the process. Another explanation could be that the antibiotic cocktail is less effective at low temperatures, as demonstrated by Germain et al. in their study on heart tissues, which showed a slight decrease in bacterial contamination after decontamination at +4°C [25]. However, a two-step decontamination procedure was effective even at low temperatures in most tissues, including CVT, suggesting that the initial bio-burden was probably very low. At any rate, the question remains whether it is advisable to perform decontamination and microbiological monitoring in two steps rather than one at the end of processing. The two-step decontamination approach, based on our methods, appears to be crucial in drastically reducing positivity at the end of the process, and this also applies to the new, more effective antibiotic cocktail. As discussed in our previous work, what probably renders the new cocktail more potent than the old one is the broader spectrum of action and the higher kill rate of its constituent antibiotics against *Streptococcus* and *Staphylococcus* species. One explanation for this higher killing activity might reside in the greater concentration of vancomycin and the presence of gentamicin, which are highly bactericidal, in place of lincomycin, which is usually considered bacteriostatic.

As highlighted by the statistical analysis, cocktail B clearly had far greater eradicating power than cocktail A considering that it reduced positive findings by 50% more than cocktail A after the first decontamination, although some tissues did remain positive at the end of the process and were therefore discarded. The change in cocktail has evidently had a beneficial effect in that less tissues were discarded than before, after deducting the tissues contaminated by non-compliers, which were discarded in any case. As part of the *in vitro* validation process, the bactericidal power of the new antibiotic cocktail was also tested at higher temperatures (as room temperature, 22°C) for shorter periods. Decontamination efficacy was found to increase (most notably allowing higher depletion of more resistant species). However, this decontamination method, which is not adopted by our institution, would have prevented us from comparing bactericidal efficacy among the various decontamination solutions.

In conclusion, targeted decontamination minimizes the risk of false negatives and, consequently, the risk of infections in recipients. In this work we compared the efficacy of two antibiotic formulations providing evidence that a cocktail including Gentamicin, Ciprofloxacin or Meropenem was more effective than the previously adopted solution composed of Ceftazidime, Lincomycin, Polymyxin B and Vancomycin, resulting in less tissues being discarded after the second decontamination step. Finally, this study highlights the need for tissue banks to systematically assess their antimicrobial cocktail to determine which one most efficiently eradicates potential pathogens from their allografts.
